# Relationship between Serum BDNF Levels and Depressive Mood in Subacute Stroke Patients: A Preliminary Study

**DOI:** 10.3390/ijms19103131

**Published:** 2018-10-12

**Authors:** Won Hyuk Chang, Min A Shin, Ahee Lee, Heegoo Kim, Yun-Hee Kim

**Affiliations:** 1 Department of Physical and Rehabilitation Medicine, Center for Prevention and Rehabilitation, Heart Vascular Stroke Institute, Samsung Medical Center, Sungkyunkwan University School of Medicine, Seoul 06351, Korea; wh.chang@samsung.com (W.H.C.); minari5995.shin@samsung.com (M.A.S.); 2 Department of Health Science and Technology, Department of Medical Device Management & Research, Department of Digital Health, SAIHST, Sungkyunkwan University, Seoul 06351, Korea; ahee.lee@gmail.com (A.L.); hiheegoo@gmail.com (H.K.)

**Keywords:** BDNF, neurotrophic factor, stroke, depression

## Abstract

The aim of this preliminary study was to investigate the potential of serum brain-derived neurotrophic factor (BDNF) as a biomarker in poststroke depressive mood in subacute stroke patients. Thirty-eight subacute stroke patients were recruited in this study. All participants underwent the standard rehabilitation program that included 2 h of physical therapy daily and 1 h of occupational therapy five days a week. The rehabilitation period lasted two weeks during the subacute stroke phase. We measured the serum BDNF, proBDNF, and matrix metalloproteinase-9 before and one and two weeks after the standard rehabilitation program. In addition, all participants were assessed using the Geriatric Depression Scale-Short Form (GDS-SF) for depressive mood at three time points. Pearson correlation analysis was performed to determine the relationship between serum BDNF levels and the GDS-SF. The GDS-SF showed significant improvement during the standard rehabilitation program period (*p* < 0.05). The GDS-SF was significantly correlated with serum BDNF levels at each time point (*p* < 0.05). These results suggest that serum BDNF may be used as a biomarker for depressive mood in subacute stroke patients. However, further studies with larger study populations are needed to clarify these results.

## 1. Introduction

Stroke is a major global cause of serious, long-term disability [1]. As the prevalence of stroke increases, the burden of disability is likely to become an increasingly important public health concern [2]. Appropriate healthcare services should be mobilized in order to reduce the burden of post-stroke disability [2]. The current best practice in stroke management is to reduce the initial impact, to take precautions to avoid further complications, and to maximize functional ability through extensive rehabilitation therapy [3]. Poststroke depression (PSD) is the most prevalent psychiatric disorder in that about one third of stroke survivors suffer from PSD, with a cumulative incidence of 55% [4]. A recent review article reported that the natural course of PSD showed a biphasic pattern, with a rise in depressive symptoms within the first six months and a new increase within the second year after stroke [5]. In addition, PSD is also known as an independent factor for mortality and functional outcome in stroke patients [6]. Therefore, many stroke rehabilitation guidelines have recommended the standardization and validation of assessment instruments to screen all stroke patients for depression in order to initiate the proper early management to improve depressive mood [7,8].

In spite of the clinical importance of PSD, early and accurate detection of depressive symptoms for stroke patients remains a challenge in clinical practice. In stroke patients with cognitive or language functional impairments, the detection of depressive mood is especially difficult. Some reports have shown that the identification of specific biomarkers may help to increase the sensitivity of PSD diagnosis. Those reports proposed the potential effectiveness of specific targeted treatment based on the pathophysiological mechanisms of PSD [4,6]. Brain-derived neurotrophic factor (BDNF) is the most abundant neurotrophin in the brain and has been reported to modulate N-methyl-D-aspartate receptor (NMDAR)-dependent long-term potentiation (LTP) and long-term depression (LTD)-related processes [9]. BDNF has often been suggested to contribute to the pathophysiology of major depressive disorder (MDD), and an important correlation between MDD and BDNF levels has been established [10]. In stroke patients, serum BDNF levels at the acute phase showed a strong relationship with the development of PSD within 3 months after onset [11]. However, the relationship between BDNF levels and depressive mood during the subacute stroke phase has not been well-studied.

Early detection and treatment of PSD can facilitate functional recovery in stroke patients and can improve the quality of life for stroke patients and their caregivers. In this preliminary study, we aimed to investigate the potential of BDNF as a biomarker in stroke rehabilitation by analyzing the relationship between serum BDNF and depressive mood in subacute stroke patients.

## 2. Results

Forty-five patients were enrolled in this study. Three patients discontinued study participation by withdrawing their consent. Among the remaining 42 patients, serum analysis in four failed due to technical problems. Data from the remaining 38 subacute stroke patients were analyzed in this study.

### 2.1. Demographic Data and Clinical Variables

The general characteristics of the 38 patients are in Table 1. The mean age of the patients was 62.95 years, the proportion of males was 60.5%, and the mean National Institutes of Health Stroke Scale (NIHSS) score at T0 was 7.5. A total of 10 (26.3%), 13 (34.2%), and 15 (39.5%) patients were allocated to the Val/Val, Val/Met, and Met/Met groups, respectively.

Twelve stroke patients could not perform the Geriatric Depression Scale-Short Form (GDS-SF) survey due to cognitive impairment. The twenty-six stroke patients who completed the GDS-SF showed a significant improvement after standard rehabilitation therapy (T2) compared with baseline (T0) (*p* < 0.05). In addition, both the NIHSS scores and the Korean Mini-Mental State Examination (K-MMSE) results in all 38 patients significantly improved at T2 compared with T0 (*p* < 0.05, Table 2).

### 2.2. Serum Levels of Mature BDNF, ProBDNF, and Metalloproteinase-9 (MMP-9)

**Figure 1:**
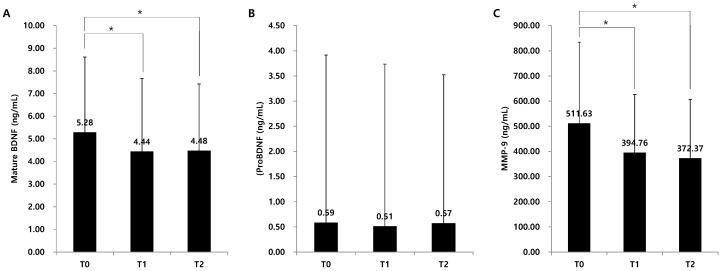
Change of serum BDNF levels. * *p* < 0.05 comparison between each time point. BDNF, brain-derived neurotrophic factor; MMP-9, matrix metalloproteinase-9. (**A**) Change of mature BDNF, (**B**) Change of proBDNF, (**C**) Change of MMP-9

Serum levels of mature BDNF were 5.81 ± 3.76 ng/mL, 4.57 ± 3.23 ng/mL, and 4.21 ± 2.66 ng/mL at T0, T1, and T2, respectively. A significant decrease at T1 and T2 compared with T0 was demonstrated (*p* < 0.05, Figure 1A). Serum levels of proBDNF were 0.59 ± 0.83 ng/mL, 0.51 ± 0.86 ng/mL, and 0.57 ± 1.08 ng/mL at T0, T1 and T2, respectively (Figure 1B). The serum levels of proBDNF did not significantly change over time. Serum levels of MMP-9 were 511.63 ± 322.64, 394.76 ± 231.46, and 372.37 ± 234.49, respectively. Similar to mature BDNF, MMP-9 significantly decreased at T1 and T2 compared with T0 (*p* < 0.05, Figure 1C).

### 2.3. Correlations with Clinical Variables

**Figure 2:**
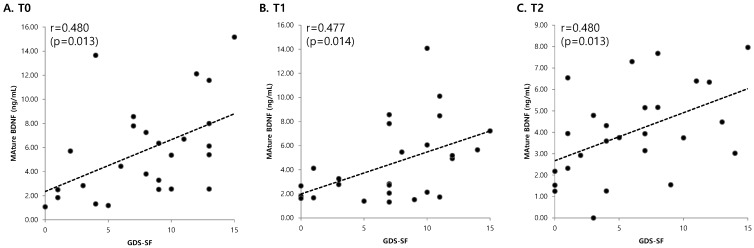
Relationships between mature BDNF levels and GDS-SF at each time point. There was a significant positive correlation between mature BDNF levels and GDS-SF at each time point. (**A**) Before the standard rehabilitation program (T0), (**B**) 1 week after the standard rehabilitation program (T1), (**C**) 2 weeks after the standard rehabilitation program (T2). BDNF, brain-derived neurotrophic factor; GDS-SF, Geriatric Depression Scale-Short Form.

Serum levels of mature BDNF showed a significant positive correlation with the GDS-SF at each time point (*p* < 0.05, Figure 2). However, K-MMSE and NIHSS results had no significant correlation with serum levels of mature BDNF. Serum levels of proBDNF were not significantly correlated with the GDS-SF, K-MMSE, and NIHSS score at any time point. Serum levels of MMP-9 at T0 showed a significant positive correlation with K-MMSE and NIHSS results (*p* < 0.05). In addition, serum levels of MMP-9 at T1 were positively correlated with the NIHSS score (*p* < 0.05, Table 3).

## 3. Discussion

This preliminary study investigated the potential role of mature BDNF in assessing depressive mood in subacute stroke patients. The results of this study revealed a significant relationship between serum mature BDNF and depressive mood in subacute stroke patients. In addition, serum levels of mature BDNF were not associated with stroke severity and cognitive function. Therefore, serum mature BDNF may be a biomarker of PSD in subacute stroke patients. However, further studies with larger participant populations will be needed to clarify these preliminary conclusions.

According to the network hypothesis, abnormalities in neural networks regulating mood is a pathophysiology of MDD [12]. BDNF has often been suggested as contributing to this pathophysiology of MDD. Serum BDNF concentrations were lower in depressive patients than in healthy controls, and the BDNF levels were significantly increased after antidepressant treatment [13]. Serum BDNF levels have also been suggested as markers for the severity of and the response to treatment of MDD [14]. Although serum BDNF may be used as a biomarker in MDD, a similar role in PSD has not been established. A previous study reported that BDNF promoter methylation status independently correlated with the prevalence, persistence, and incidence of PSD, as well as with the worsening of depression severity over a one-year period after stroke [15]. In addition, a recent meta-analysis showed that the decrease in serum BDNF concentrations in early stroke predisposed patients to the development of PSD [16]. However, in this study with subacute stroke patients, serum levels of mature BDNF showed a positive correlation with depressive mood. Although these results may differ from the previous study [16], the different phases in which depressive mood was assessed could have affected these results. In contrast with the previous study, the assessments of depressive mood were performed at the same time as analysis of serum BDNF levels. Therefore, the follow-up assessments for depressive mood at the chronic stroke phase are needed to clarify the role of serum mature BDNF in PSD.

Mature BDNF has a pronounced protective and neurotrophic effect, and brain mature BDNF has been shown to increase after stroke [17]. Aerobic exercise promotes changes in central BDNF concentrations post-stroke in animal models and increased serum BDNF concentration in post-stroke patients [18]. According to these reports, BDNF has emerged as a key facilitator of neuroplasticity for motor learning and rehabilitation in stroke [19]. In addition, low levels of serum BDNF concentrations at the acute phase were associated with poor functional outcome at two years after stroke [20]. Therefore, serum levels of mature BDNF may correlate with functional levels in stroke patients. However, in this study, stroke severity and cognitive function before standard rehabilitation therapy were associated with MMP-9, not serum mature BDNF. MMP-9 plays a key role in synaptic plasticity of the brain and acts by converting proBDNF to mature BDNF [21]. MMP-9 may have more important roles in Val/Met and Met/Met BDNF genotypes than in Val/Val because the single nucleotide polymorphism at nucleotide 196G/A affects intracellular processing and secretion of BDNF [22]. The relationship of MMP-9 with stroke severity and cognitive function in this study may be due to the relatively large number of stroke patients with the Met allele. However, we could not assess the serum levels of mature BDNF, proBDNF, and MMP-9 according to BDNF genotype because of the relatively small number of patients. Therefore, another study with a larger number of patients is needed.

This study had some limitations. First, because the survey for depressive mood requires higher cognitive and language functions, 12 subacute stroke patients in this study could not perform the survey. This may have biased the study; however, biomarkers in PSD can disprove this possibility. In addition, we could not assess depressive mood at the chronic stroke phase nor could we correct for age, sex, stroke severity, lesion characteristics, and current medication. Particularly, we could not consider the relationship between serum BDNF levels and various functional impairments such as motor and cognition in stroke patients. Therefore, further study with a larger number of stroke patients is needed in the future. In spite of these limitations, the results of this study suggest that serum BDNF may be used as a biomarker for depressive mood in subacute stroke patients.

## 4. Materials and Methods

### 4.1. Participants

Subacute hemiplegic inpatient stroke patients (less than one month from onset) were included in the study. Subacute stroke phase in this study was defined from 1 week to 3 months after onset [8]. Exclusion criteria were: (1) progressive or unstable stroke, (2) pre-existing or active major neurological disease or major psychiatric disease, (3) a history of significant alcohol or drug abuse within the last three years, and (4) advanced liver, kidney, cardiac or pulmonary disease. Written informed consent was obtained from all subjects prior to inclusion in the study, and the study protocol was approved by the Samsung Medical Center Institutional Review Board (SMC 2016-08-059, 26 September 2016).

### 4.2. Experimental Design

This study was a longitudinal observational study during the standard inpatient rehabilitation therapy period. All participants underwent the standard rehabilitation program that included 2 h of physical therapy daily and 1 h of occupational therapy 5 days a week. The inpatient rehabilitation period was 2 weeks during the subacute stroke phase. We measured serum BDNF, proBDNF and matrix MMP-9 at T0 (before the standard rehabilitation program), T1 (1 week after the standard rehabilitation program), and T2 (2 weeks after the standard rehabilitation program). In addition, all participants were assessed for functional impairments and depressive mood at each time point.

### 4.3. Measurement of Mature BDNF, ProBDNF, and MMP-9 Serum Levels

Serum levels of mature BDNF, proBDNF, and MMP-9 were measured using the human BDNF ELISA Kit (Adipo Bioscience, Santa Clara, CA, USA), the human proBDNF ELISA Kit (Adipo Bioscience), and the human MMP-9 ELISA Kit (R&D Systems, Minneapolis, MN, USA), respectively. The optical density of each well was measured using an automated microplate reader (Emax; Molecular Devices, Sunnyvale, CA, USA) [21].

### 4.4. BDNF Genotyping Technique

Whole blood was collected into EDTA tubes. Genomic DNA was isolated from peripheral blood leukocytes according to a standard proteinase-K RNase digestion procedure followed by phenol-chloroform extraction. The BDNF Val66Met polymorphism was genotyped via PCR-RFLP [22].

### 4.5. Assessment of Clinical Variables

At each time point, depressive mood was assessed using the GDS-SF (range: 0–15) [23]. The GDS-SF is a subset of 15 questions from the original Geriatric Depression Scale well-validated for detection of depressive symptoms in stroke patients [24]. In addition, functional assessments for the severity of stroke were performed using the NIHSS (range: 0–42) [21]. Cognitive function was assessed using the K-MMSE (range: 0–30) [25]. Cognitive function was assessed using the K-MMSE (range: 0–30) [25].

### 4.6. Data Analysis

Statistical analyses used SPSS/PC + software 25.0 version (SPSS Inc., Chicago, IL, USA). A paired *t*-test with Bonferroni’s correction was used to compare values between each time point. Pearson correlation analysis was used to assess the relationships between serum mature BDNF, proBDNF, and MMP-9 levels and clinical variables. *p*-Values less than 0.05 were considered statistically significant.

## Figures and Tables

**Table 1:** General characteristics of patients.

Parameters	Total Patients (*n* = 38)	Patients Who Performed GDS-SF (*n* = 26)
Sex (male:female)	23:15	19:7
Age	62.9 ± 14.6 (28–86)	59.9 ± 13.2 (33–86)
Stroke type (infarct:hemorrhage)	27:11	17:9
Supratentorial:Intratentorial	35:3	23:3
Lesion side (right:left)	18:20	17:9
Duration of stroke (T0, days)	15.8 ± 6.0 (6–28)	16.0 ± 5.7 (6–28)
NIHSS at T0	7.5 ± 5.4 (0–22)	5.8 ± 4.3 (0–14)
BDNF genotype (Val/Val:Val/Met:Met/Met)	10:13:15	6:10:10

GDS-SF, Geriatric Depression Scale-Short Form; NIHSS, National Institutes of Health Stroke Scale; BDNF, brain-derived neurotrophic factor.

**Table 2:** Change of clinical variables.

Parameters	T0	T1	T2
GDS-SF (*n* = 26)	7.9 ± 4.4	7.2 ± 4.5	6.0 ± 4.6 *
NIHSS (*n* = 38)	7.5 ± 5.4	6.8 ± 4.7	6.6 ± 4.8 *
K-MMSE (*n* = 38)	16.9 ± 12.3	18.0 ± 12.7	18.7 ± 12.6 *

Values are presented as mean ± standard deviation. GDS-SF, Geriatric Depression Scale-Short Form; NIHSS, National Institutes of Health Stroke Scale; K-MMSE, Korean Mini-Mental Status Examination. * *p* < 0.05 compared with T0.

**Table 3:** Correlation analysis of serum levels of BDNF and clinical variables.

Parameters		GDS-SF (*n* = 26)			K-MMSE (*n* = 38)			NIHSS (*n* = 38)		
		T0	T1	T2	T0	T1	T2	T0	T1	T2
Mature BDNF (ng/mL)	T0	0.480 *	-	-	0.043	-	-	0.045	-	-
		(0.013)			(0.800)			(0.788)		
	T1	-	0.477 *	-	-	−0.104	-	-	0.186	-
			(0.014)			(0.541)			(0.263)	
	T2	-	-	0.480 *	-	-	−0.180	-	-	0.194
				(0.013)			(0.280)			(0.243)
ProBDNF (ng/mL)	T0	0.006	-	-	−0.168	-	-	0.126	-	-
		(0.977)			(0.313)			(0.452)		
	T1	-	0.141	-	-	−0.111		-	0.117	-
			(0.492)			(0.513)			(0.483)	
	T2	-	-	0.373	-	-	0.048	-	-	0.024
				(0.060)			(0.775)			(0.888)
MMP-9 (ng/mL)	T0	0.079	-	-	−0.394 *	-	-	0.495 *	-	-
		(0.700)			(0.014)			(0.002)		
	T1	-	0.312	-	-	−0.191		-	0.355 *	-
			(0.120)			(0.256)			(0.029)	
	T2	-	-	0.093	-		−0.207	-	-	0.147
				(0.651)			(0.213)			(0.379)

Values are presented as correlation coefficient (*p*-value). GDS-SF, Geriatric Depression Scale-Short Form; K-MMSE, Korean Mini-Mental Status Examination; NIHSS, National Institutes of Health Stroke Scale; BDNF, brain-derived neurotrophic factor; MMP-9, matrix metalloproteinase-9. * *p* < 0.05.
